# Cachexia Index and CRP–Triglyceride–Glucose Index as Prognostic Predictors in Cancer Hospitalized Patients

**DOI:** 10.3390/nu18213705

**Published:** 2026-11-08

**Authors:** Pablo Lozano-Martínez, Rosana Urdániz Borque, Gema González Fernández, Clara Fernández Rivera, Alba Gil-Pascual, Carlota Losfablos de Castro, José Antonio Gimeno Orna, Julia Ocón Bretón

**Affiliations:** 1 Service of Endocrinology and Nutrition, Hospital Clínico Universitario Lozano Blesa, 50009 Zaragoza, Spain; 2 Instituto de Investigación Sanitaria de Aragón (IIS Aragón), 50009 Zaragoza, Spain

**Keywords:** Cachexia Index, CRP–triglyceride–glucose index, cancer cachexia, body composition, prognostic index

## Abstract

Background/Objectives: Cancer cachexia is linked to poor oncological outcomes. The Cachexia Index (CXI) and the CRP–triglyceride–glucose index (CTI), composite markers of body composition, nutrition, and inflammation, have emerged as prognostic tools, but their value in hospitalized cancer patients remains unexplored. We evaluated their association with short- and long-term outcomes in this population. Methods: This retrospective, observational study included 139 hospitalized patients with active cancer by a Nutrition Unit during 2023. Nutritional status was assessed using the Global Leadership Initiative on Malnutrition (GLIM) criteria; body composition was quantified by computed tomography (CT) imaging at L3 level. Primary outcomes were in-hospital mortality, intensive care unit (ICU) admission due to clinical deterioration, hospital readmission, and overall survival. Each index was analyzed as a continuous variable, in quartiles, by the median, and at an exploratory ROC-derived cut-off, using logistic and Cox regression. Results: CXI at its ROC-derived cut-off (<9.03; AUC 0.69) was associated with in-hospital mortality (OR 3.91; 95% CI 1.22–12.52; *p* = 0.022) and overall survival (HR 1.75; 95% CI 1.04–2.95; *p* = 0.035), unlike its continuous, quartile, and median forms. CTI at its cut-off (≥6.71; AUC 0.704) remained associated with ICU admission after adjustment for surgery and age (OR 5.52; 95% CI 1.64–18.54; *p* = 0.006) or surgery and myosteatosis (OR 4.94; 95% CI 1.48–16.47; *p* = 0.009), but not with survival. Conclusions: CXI and CTI show complementary, threshold-dependent prognostic value: CXI identifies in-hospital and long-term mortality risk, and CTI, more consistently than CXI, identifies ICU admission risk due to clinical deterioration. These exploratory findings need prospective, multicenter validation.

## 1. Introduction

Cancer cachexia is a multifactorial metabolic syndrome characterized by an ongoing loss of skeletal muscle mass—with or without loss of fat mass—that cannot be fully reversed by conventional nutritional support and leads to progressive functional impairment [1]. According to the international consensus definition proposed by Fearon and colleagues, the diagnosis requires a weight loss greater than 5% over the preceding six months, or greater than 2% in patients with a body mass index (BMI) below 20 kg/m^2^ or with pre-existing sarcopenia, and is further staged into pre-cachexia, cachexia, and refractory cachexia based on the degree of metabolic derangement and therapeutic responsiveness [1]. Despite the clarity of this framework, its application in routine clinical practice remains challenging, as weight loss alone is an insensitive marker that may be masked by fluid retention or obesity, and fails to capture the underlying loss of metabolically active tissue that drives the prognostic impact of the syndrome [2,3].

This has prompted growing interest in the incorporation of objective body composition parameters—particularly those derived from cross-sectional imaging—as essential components of cachexia assessment, moving beyond weight-based criteria towards a more precise characterization of the cachectic phenotype [4]. Its clinical consequences extend well beyond muscle wasting, encompassing increased postoperative morbidity, longer hospital stays, higher rates of treatment discontinuation, and poorer overall survival [5,6], making early and accurate identification of at-risk patients a priority in the nutritional management of the oncological population.

Nutritional status has long been recognized as an independent determinant of prognosis in cancer patients [7,8,9]. Beyond classical anthropometric and biochemical parameters, body composition assessment by computed tomography (CT) has emerged as a valuable tool for the objective quantification of skeletal muscle mass and quality. Low skeletal muscle index (SMI) and myosteatosis have both been independently associated with adverse clinical outcomes in oncological populations [4]. These findings have driven interest in the development of composite indices capable of integrating body composition data with other prognostically relevant variables, with the aim of capturing the complexity of the cachectic phenotype in a single, reproducible measure.

The Cachexia Index (CXI) is one such composite tool, combining the SMI derived from CT imaging at the level of the third lumbar vertebra (L3), serum albumin concentration, and the neutrophil-to-lymphocyte ratio (NLR)—three parameters that jointly reflect the interplay between muscle wasting, nutritional depletion, and systemic inflammation that underlies cancer cachexia. A growing body of evidence has demonstrated its prognostic value across multiple tumor types and clinical settings, with low CXI consistently associated with reduced survival and increased morbidity [10,11].

The CRP–triglyceride–glucose index (CTI) represents a complementary approach, combining C-reactive protein (CRP) levels with the triglyceride–glucose index (TyG) as an integrated measure of systemic inflammation and insulin resistance. The index was originally proposed and validated by Ruan and colleagues, who demonstrated its independent prognostic value across multiple tumor types, with higher CTI values associated with significantly reduced overall survival [12]. Subsequent work by the same group further confirmed its relevance in patients with cancer cachexia specifically, where elevated CTI was associated with worse nutritional status and poorer clinical outcomes [13], supporting its potential utility as a marker of metabolic deterioration in the oncological setting.

The present study aimed to evaluate the association of CXI and CTI with short- and long-term clinical outcomes—including in-hospital mortality, intensive care unit (ICU) admission, hospital readmission, and overall survival—in a cohort of cancer patients admitted to medical and surgical wards. To our knowledge, this represents one of the first studies to examine both indices simultaneously in this clinical setting, using standardized nutritional assessment tools and CT-based body composition analysis as part of routine clinical care.

## 2. Materials and Methods

Data was collected for this retrospective, observational study including patients evaluated by the Nutrition Unit at Hospital Clínico Universitario Lozano Blesa in Zaragoza, Spain during January–December 2023. Consecutive patients meeting the inclusion criteria were identified through the electronic medical records of the hospital. Clinical, anthropometric, analytical and imaging data were collected retrospectively from routinely recorded information in the electronic medical record system.

The study was conducted in accordance with the principles of the Declaration of Helsinki. Approval was obtained from the Comité de Ética de la Investigación de Aragón (CEICA) on November 2024, approval code C.I. PI24/433. Due to the retrospective nature of the study and the use of pseudonymized data, the requirement for informed consent was not required. The study is reported in line with the STROBE guidelines for observational studies.

### 2.1. Patients

The inclusion criteria were as follows: (1) patients aged ≥18 years who were hospitalized in medical or surgical wards with a diagnosis of active cancer and evaluated by the Nutrition Unit at Hospital Clínico Universitario Lozano Blesa, Zaragoza, Spain; (2) availability of a CT scan including the abdominal region at the L3 level, performed within 30 days before or after the nutritional assessment; and (3) availability of all required laboratory data to calculate the CTI and CXI.

Exclusion criteria included (1) lack of a minimum follow-up of at least one year from the time of data collection, unless follow-up was completed by death, and (2) patients admitted in a critical care setting during the evaluation. After applying these criteria, a total of 139 patients were included in the final analysis.

### 2.2. Outcomes

Primary outcomes included ICU admission due to clinical deterioration, in-hospital mortality, hospital readmissions, and overall survival during follow-up period. Hospital readmission was analyzed only among patients discharged alive.

The objective of this study was to evaluate the association of the cachexia-related indices CTI and CXI with the primary outcomes. In addition, the associations between body composition parameters (skeletal muscle mass, muscle radiodensity), nutritional status, and CTI and CXI were evaluated.

### 2.3. Study Variables

Baseline data collected included age, sex, cancer type and stage, oncological treatment modalities (surgery, chemotherapy, immunotherapy and/or radiotherapy), nutritional assessment information and blood test results.

### 2.4. Nutritional Assessment

A comprehensive nutritional assessment was performed as part of routine clinical care. Anthropometric variables collected included height, body weight, unintentional weight loss, and BMI. The risk of malnutrition was evaluated using the Malnutrition Universal Screening Tool (MUST), and the diagnosis of malnutrition was established according to Global Leadership Initiative on Malnutrition (GLIM) criteria. When available, the risk of sarcopenia was additionally screened using the SARC-F questionnaire, as part of the standard nutritional evaluation.

### 2.5. Body Composition Analysis

Body composition was assessed using CT images at L3, an anatomical landmark validated for the estimation of skeletal muscle and adipose tissue compartments. Cross-sectional areas (cm^2^) of skeletal muscle from the selected images were analyzed using the FocusedOn software (Las Palmas de Gran Canaria, Spain; https://focusedon.es, accessed on 2 December 2024). from ARTIS Development.

To account for inter-individual differences in body size, the skeletal muscle cross-sectional area at L3 was normalized for height squared (m^2^), resulting in the SMI, expressed as cm^2^/m^2^.

Muscle quality was further evaluated by assessing skeletal muscle radiodensity, defined as the mean radiological attenuation of muscle tissue within the region of interest and expressed in Hounsfield Units (HU). Lower radiodensity values are indicative of intramuscular fat infiltration (myosteatosis).

Low skeletal muscle mass was defined using sex-specific cut-off values of ≤39 cm^2^/m^2^ for women and ≤55 cm^2^/m^2^ for men [14]. Myosteatosis was defined according to previously established radiodensity thresholds: <41 HU for patients with BMI < 25 kg/m^2^ and <33 HU for those with BMI ≥ 25 kg/m^2^ [4].

### 2.6. Laboratory Data and Derived Indices

Laboratory parameters obtained at the time of nutritional assessment included serum CRP, hemoglobin, albumin, triglycerides, fasting glucose, serum creatinine and other routinely collected biochemical variables. These parameters were used to calculate inflammatory, metabolic and cachexia-related indices, which are described in detail in the following section.

### 2.7. Cachexia Index (CXI)

The CXI was calculated according to previously published methodology [10], using the following formula:

CXI = SMI × serum albumin (g/dL)/NLR

### 2.8. CRP–Triglyceride–Glucose Index (CTI)

The CTI was calculated using the following equation, as proposed in previous bibliography [12]:

CTI = 0.412 × ln(CRP) + TyG

where the triglyceride–glucose index (TyG) was defined as

TyG = ln(triglycerides × fasting glucose)/2.

CRP levels were expressed in mg/L, and fasting glucose and triglyceride concentrations were expressed in mg/dL.

### 2.9. Statistical Analysis

Continuous variables were summarized as mean and standard deviation (SD) when normally distributed, and as median and interquartile range (IQR) when non-normally distributed. Normality was assessed using the Kolmogorov–Smirnov test and by visual inspection of histograms and normal probability plots. Categorical variables were described as absolute frequencies and percentages.

An initial descriptive and comparative analysis were performed to explore the association between CTI, CXI and clinical outcomes. Comparisons of continuous variables between two groups were performed using the independent-samples t-Student test for normally distributed variables or the Mann–Whitney U test for non-normally distributed variables. Comparisons of categorical variables were conducted using the χ^2^ test or Fisher’s exact test, as appropriate.

Receiver operating characteristic (ROC) curve analyses were performed to evaluate the discriminatory ability of the indices in relation to in-hospital mortality and ICU admission. The area under the curve (AUC) was calculated along with 95% confidence intervals (95% CI). Optimal cut-off values were determined using the Youden index.

Subsequently, univariate regression analyses were conducted to identify variables associated with outcomes. Multivariable logistic regression models were developed to identify independent predictors of the outcomes. Variables showing statistical significance in univariable analyses, as well as those considered clinically relevant, were included in the multivariable models. Results were reported as odds ratios (OR) with corresponding 95% CI. Multivariable models were fitted on complete cases, and the number of patients and events included in each model is reported; patients with missing tumor stage were excluded from the models that included this variable.

Survival was analyzed using Kaplan–Meier estimates, with differences between groups assessed by the log-rank (Mantel–Cox) test. Analyses were performed according to CXI and CTI, considered both as categorical variables (quartiles and median-based dichotomization) and as continuous measures.

Cox proportional hazards regression models were subsequently used to assess independent associations with overall survival, reporting hazard ratios (HR) and 95% CI. The proportional hazards assumption was verified prior to model interpretation. Main associations were also assessed separately in digestive and other tumors, and heterogeneity was tested with index × tumor group interaction terms.

A two-sided *p* value < 0.05 was considered statistically significant. All statistical analyses were performed using IBM SPSS Statistics (version 25; IBM Corp., Armonk, NY, USA).

Prior to statistical analyses, a predefined data-cleaning procedure was applied to identify and manage outliers. In approximately normally distributed quantitative variables, univariate outliers were identified using standardized z scores, with extreme values defined as |z| > 3.0. For variables showing relevant skewness, Tukey’s method was applied, excluding only extreme outliers beyond Q1 − 3 × IQR or Q3 + 3 × IQR, while mild outliers (1.5–3 × IQR) were retained in the analysis.

### 2.10. GenAI Use

Generative artificial intelligence tools were used to assist with the drafting and editing of the manuscript text. These tools were not used for data collection, data analysis, statistical analysis, or figure generation. All scientific content, analyses, and interpretations were performed by the authors.

## 3. Results

A final cohort of 139 patients was included in the study. Full baseline characteristics of the population of study are summarized in Table S1, available in Supplementary Material.

All patients had complete data available for the calculation of the CTI and CXI, as well as information on in-hospital mortality, ICU admission, hospital readmissions, and overall survival during follow-up.

The mean age of the cohort was 65.1 ± 12.7 years, and 73 patients (52.5%) were male. Median BMI was 24.0 kg/m^2^ (IQR 21.1–26.6).

Regarding nutritional status, 98 patients (79.0%) met the criteria for disease-related malnutrition according to the GLIM definition. Among these patients, malnutrition was classified as severe in 57 cases (58.2%).

In terms of body composition, the mean SMI was 40.5 ± 8.9 cm^2^/m^2^ and mean skeletal muscle radiodensity was 33.5 ± 13.0 HU. Based on the predefined cut-off values, 111 patients (79.9%) were classified as having low skeletal muscle mass, and 94 patients (67.6%) met criteria for myosteatosis on CT images at the L3 level.

### 3.1. Comparison of Clinical Characteristics by CXI and CTI Quartiles

To explore relationships across the full range of both indices, the cohort was stratified into quartiles of CXI and CTI. *p* for trend was calculated using linear regression for continuous variables and using the linear-by-linear association chi-square test for categorical variables.

#### 3.1.1. CXI Quartiles

Full results on baseline characteristics according to CXI quartiles are summarized in Table S2A available in Supplementary Materials. Higher CXI quartiles were associated with progressively better body composition parameters. Serum albumin increased while CRP declined inversely (*p* trend < 0.001). Hemoglobin, skeletal muscle index, and muscle radiodensity all showed significant positive trends.

Oncological characteristics did not differ significantly across quartiles. Surgery was more frequent in lower CXI quartiles (82.4% in Q1 vs. 42.9% in Q4; *p* trend < 0.001), while chemotherapy showed the opposite pattern (58.8% vs. 82.9%; *p* = 0.031). Myosteatosis prevalence decreased significantly with increasing CXI (79.4% vs. 54.3%; *p*-trend = 0.023), while malnutrition and sarcopenia did not differ significantly.

Regarding clinical outcomes, in-hospital mortality declined significantly across quartiles (20.6% in Q1 vs. 5.7% in Q4; *p*-trend = 0.029). Among patients discharged alive (n = 123), hospital readmission did not differ significantly across CXI quartiles (59.3%, 60.0%, 57.6% and 81.8% in Q1–Q4; *p*-trend = 0.083). ICU admission also declined across quartiles (26.5%, 14.3%, 11.4% and 2.9% in Q1–Q4; *p*-trend = 0.005), whereas overall mortality showed no trend (*p* = 0.785).

#### 3.1.2. CTI Quartiles

Full results on baseline characteristics according to CTI quartiles are summarized in Table S2B available in Supplementary Materials. CTI quartiles were defined by a stepwise increase in the index from 5.31 ± 0.51 in Q1 to 7.29 ± 0.21 in Q4. As expected from its construction, the steepest gradients were observed for CRP and TyG index (*p* < 0.001). Albumin declined inversely across quartiles (*p* < 0.001) and hemoglobin likewise (*p* = 0.009), and NLR increased progressively (*p* = 0.001). Muscle radiodensity decreased significantly with higher CTI (*p* = 0.009), while SMI showed a positive trend (*p* = 0.039).

Tumor characteristics were homogeneously distributed across CTI quartiles, with no significant trends. Surgery was significantly more frequent in higher CTI quartiles (*p* trend = 0.003).

The most relevant clinical gradient was observed for ICU admission, which increased from 5.9% in Q1 to 25.7% in Q4 (*p*-trend = 0.007). Among patients discharged alive, hospital readmission showed no significant trend across CTI quartiles (80.0% in Q1 vs. 50.0% in Q4; *p*-trend = 0.059). In-hospital mortality showed a non-significant upward trend across quartiles (11.8% in Q1 vs. 25.7% in Q4; *p* = 0.063), and overall mortality did not differ significantly (*p* = 0.302).

### 3.2. Association of CXI and CTI with Clinical Outcomes (Continuous, Quartile, and Median Forms)

CXI and CTI were further compared across ICU admission, in-hospital mortality, and overall mortality, entering each index as a continuous variable, in quartiles, and dichotomized by the median. Results are summarized in Table 1.

Regarding ICU admission, both indices were associated with the outcome in all three forms. Patients admitted to the ICU had lower continuous CXI values (6.48 vs. 14.66; *p* = 0.002), a significant quartile trend (*p* = 0.005) and a higher proportion of low-CXI patients (73.7% vs. 45.8%; *p* = 0.024). They also had higher continuous CTI values (7.02 vs. 6.54; *p* = 0.004), a significant quartile trend (*p* = 0.007) and a higher proportion of high-CTI patients (78.9% vs. 45.8%; *p* = 0.007). Full data about ICU admission are available in Table S3 in Supplementary Materials.

For in-hospital mortality, CXI was significantly associated across all three forms: lower continuous CXI values were observed in patients who died (6.59 vs. 14.65; *p* = 0.013), with a significant quartile trend (*p* = 0.029) and a higher proportion of low-CXI patients among non-survivors (75.0% vs. 47.2%; *p* = 0.036). CTI showed no significant association with in-hospital mortality in its continuous or median form (*p* = 0.095 and *p* = 0.104, respectively); the quartile distribution suggested a possible non-linear pattern, but this should be interpreted with caution given the low number of in-hospital deaths (N = 16). Full data about in-hospital mortality are available in Table S4 in Supplementary Materials.

Neither CXI nor CTI, in any of the three forms, showed a significant association with overall mortality. Full data about overall mortality are available in Table S5 in Supplementary Materials.

### 3.3. ROC Curve Analysis and Optimal Cut-Off Points

**Figure 1:**
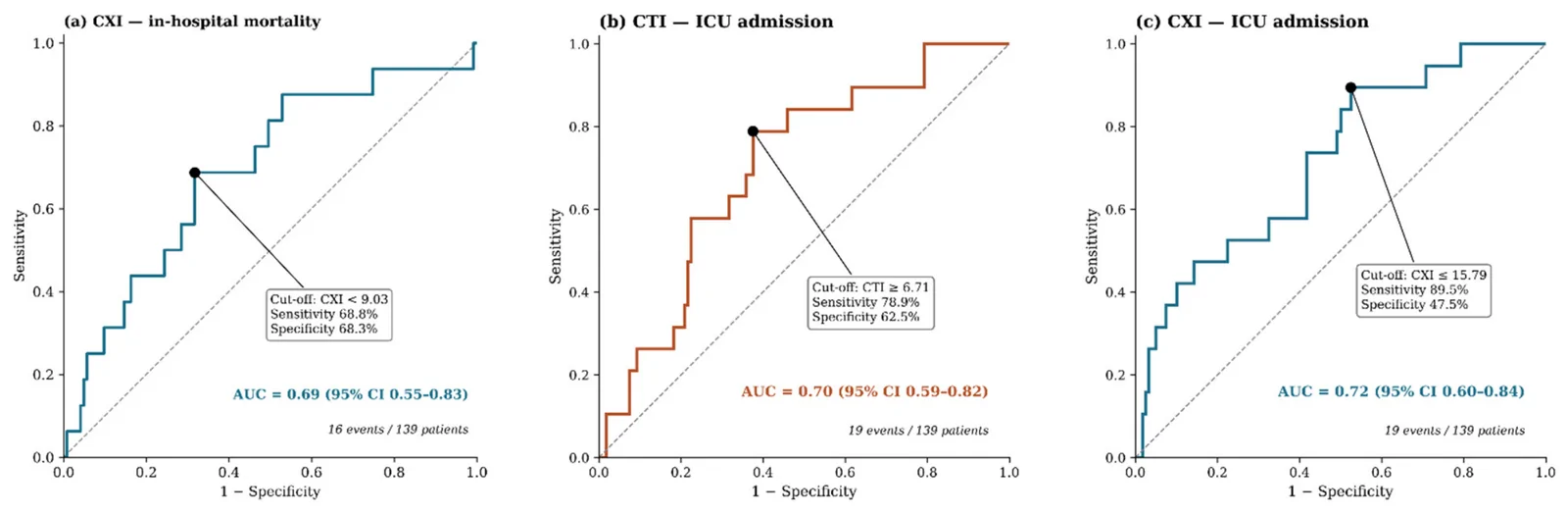
ROC curves of the CXI for in-hospital mortality (**a**) and of the CTI (**b**) and CXI (**c**) for ICU admission due to clinical deterioration. The area under the curve (AUC) with its 95% confidence interval is shown in each panel. The black dot marks the cut-off point selected with the Youden index (CXI < 9.03; CTI ≥ 6.71; CXI ≤ 15.79), with its sensitivity and specificity. Classificatory tables with dimensions of 2 × 2 are provided in Table S6.

Given the significant associations identified between CXI and in-hospital mortality, and between both indices and ICU admission, ROC curve analysis was restricted to these three index–outcome pairs (Figure 1). Classificatory tables with dimensions of 2 × 2 are provided in Table S6.

For in-hospital mortality, CXI showed an AUC of 0.69 (95% CI 0.55–0.83), and the optimal cut-off value of <9.03 defined low CXI. Patients below this threshold had a significantly increased risk of in-hospital mortality (OR 4.74; 95% CI 1.54–14.57), with a sensitivity of 68.8% and a specificity of 68.3%.

For ICU admission, CTI had an AUC of 0.704 (95% CI 0.588–0.819), and the cut-off of ≥6.71 defined high CTI (OR 6.25; 95% CI 1.95–20.00; sensitivity 78.9%, specificity 62.5%). CXI had an AUC of 0.721 (95% CI 0.601–0.840), and the cut-off of ≤15.79 defined low CXI (OR 7.69; 95% CI 1.70–34.76; sensitivity 89.5%, specificity 47.5%). This CXI cut-off is close to the cohort median (13.56) and classified 57.6% of the patients as low CXI. All cut-offs were derived in the same sample and should be considered exploratory.

### 3.4. Multivariable Logistic Regression for CXI and In-Hospital Mortality

To assess the independent association between CXI and in-hospital mortality, backward Wald logistic regression models were constructed for each of the four analytical forms of CXI (continuous, quartiles, median split, and the ROC-derived cut-off). Given the limited number of in-hospital deaths (N = 16), two independent adjustment sets were tested—age + tumor stage and myosteatosis + tumor stage. Tumor stage was missing in 15 patients, who were excluded from these models; each model therefore included 124 patients and 14 deaths. Results are summarized in Table 2, and the estimates of all variables entered in each model are provided in Table S7.

In all models, age, myosteatosis and tumor stage were eliminated (*p* ≥ 0.38 in the full models). CXI as a continuous variable, in quartiles and as a median split was also eliminated, so the final models contained no variable.

CXI dichotomized at the ROC-derived cut-off (<9.03) was the only form retained, and it remained alone in the final model under both candidate sets, which were therefore identical (OR 3.91; 95% CI 1.22–12.52 in both; *p* = 0.022; Nagelkerke R^2^ = 0.086). In the 139 patients with complete CXI and outcome data, the unadjusted OR for the cut-off was 4.74 (95% CI 1.54–14.57; *p* = 0.007).

### 3.5. Multivariable Logistic Regression for CTI, CXI and ICU Admission

To assess the relationship of CTI and CXI with ICU admission, backward Wald logistic regression models were built for each of the four analytical forms of each index, with the index entered together with the covariates and eligible for elimination. Given the limited number of events (N = 19), two adjustment sets were tested: surgery + age and surgery + myosteatosis. Surgery was included in both because 18 of the 19 events occurred in operated patients. Age and myosteatosis were selected from the univariable analysis (*p* = 0.062 and *p* = 0.034, respectively) and on clinical grounds. Results are shown in Table 3, and the estimates of all variables entered in each model are given in Table S8.

CTI was retained in the final model in its continuous, median-split and ROC-derived forms under both adjustment sets, whereas CTI quartiles were eliminated (global *p* = 0.169 and 0.268). At the ROC-derived cut-off (≥6.71), CTI was significantly associated with ICU admission under both sets (OR 5.52; 95% CI 1.64–18.54; *p* = 0.006 and OR 4.94; 95% CI 1.48–16.47; *p* = 0.009). The median split was significant with adjustment 1 (OR 3.43; 95% CI 1.03–11.43; *p* = 0.045) but not with adjustment 2 (OR 3.14; 95% CI 0.94–10.49; *p* = 0.063). The continuous form did not reach significance.

CXI was eliminated as a continuous variable, in quartiles and as a median split under both sets (*p* ≥ 0.144 in the full models). Only the ROC-derived cut-off (≤15.79) was retained in both sets, with similar estimates (OR 5.45; 95% CI 1.18–25.31; *p* = 0.030 with adjustment 1, and OR 4.56; 95% CI 0.96–21.57; *p* = 0.056 with adjustment 2).

Surgery was retained in every final model (OR 6.5–11.1; *p* = 0.022–0.077), with wide confidence intervals because only one event occurred in a non-operated patient. In the 95 operated patients (18 events), the CTI cut-off remained associated with the outcome (OR 4.67; 95% CI 1.41–15.48; *p* = 0.012), and CXI showed no consistent association. Given the small number of events, these models are exploratory and the cut-offs were derived in the same sample.

### 3.6. Sensitivity Analysis: Kaplan–Meier Survival Analysis

**Figure 2:**
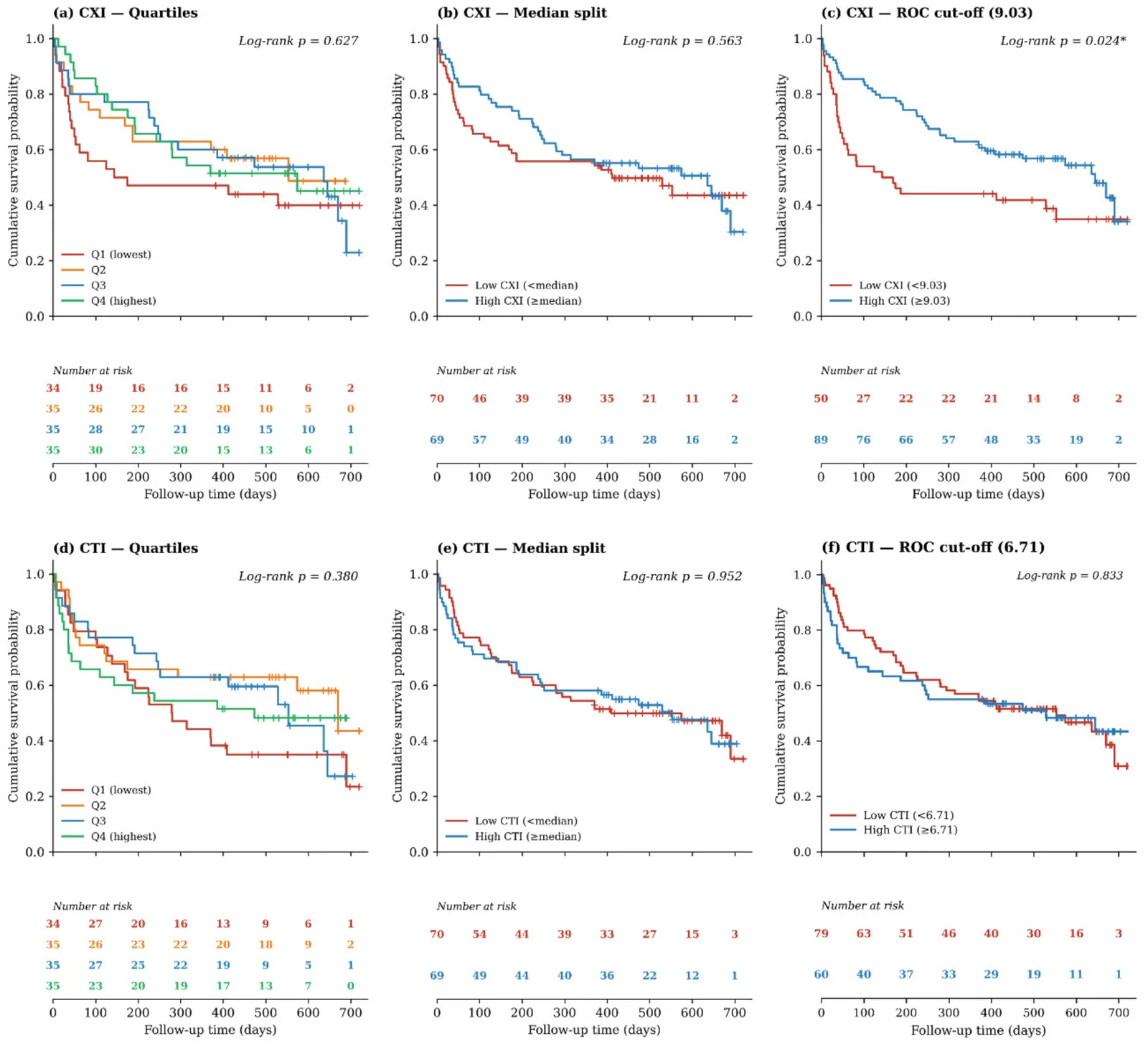
Kaplan–Meier curves for overall survival according to CXI and CTI. (**a**) Overall survival stratified by CXI quartiles (Q1–Q4). (**b**) Overall survival stratified by CXI median split (low vs. high). (**c**) Overall survival stratified by the ROC-derived CXI cut-off (<9.03 vs. ≥9.03). (**d**) Overall survival stratified by CTI quartiles (Q1–Q4). (**e**) Overall survival stratified by CTI median split (low vs. high). (**f**) Overall survival stratified by the ROC-derived CTI cut-off (<6.71 vs. ≥6.71). Cumulative survival probability is shown on the Y-axis and follow-up time (days) on the X-axis. Tick marks (+) indicate censored observations. Only the ROC-derived CXI cut-off (**c**) showed a statistically significant difference in overall survival (log-rank *p* = 0.024); no significant differences were observed for any other analytical form of CXI or CTI (log-rank *p* > 0.05, panels (**a**–**f**)). Numbers at risk are shown below each panel. * shows *p* < 0.05.

Kaplan–Meier survival analysis was performed as a sensitivity analysis to assess whether the associations observed between CXI, CTI, and acute clinical outcomes (in-hospital mortality, ICU admission) extended to overall survival during follow-up (Table 4, Figure 2). The cohort included 139 patients, with 74 deaths recorded (53.2%) and a median follow-up of 553 days.

For CXI, no significant differences in overall survival were observed when patients were stratified by quartiles or by the median. However, when CXI was dichotomized using the ROC-derived cut-off (<9.03), a significant difference in overall survival emerged: patients with low CXI had a markedly shorter median survival than those with high CXI (144 vs. 645 days; log-rank χ^2^ = 5.130, *df* = 1; *p* = 0.024).

For CTI, no significant differences in overall survival were observed across any of the three analytical forms.

### 3.7. Cox Proportional Hazards Regression for Overall Survival

Cox univariate regression was performed for CXI and CTI in their four analytical forms (continuous, quartiles, median split, and ROC-derived cut-off), together with candidate clinical covariates. Age showed a borderline association with overall mortality (HR 1.017; 95% CI 0.998–1.036; *p* = 0.075), while malnutrition, advanced tumor stage, and surgery were all strongly associated with overall mortality in the expected clinical direction (all *p* ≤ 0.001). Neither CXI nor CTI showed a significant univariate association with overall mortality in their continuous, quartile, or median forms. Notably, CXI dichotomized at the ROC-derived cut-off (<9.03) was the only form of either index significantly associated with overall mortality (HR 0.589; 95% CI 0.371–0.936; *p* = 0.025 for high vs. low CXI), consistent with the Kaplan–Meier findings described above.

Multivariable Cox regression models with backward stepwise elimination (likelihood ratio) were subsequently constructed for each of the eight index–form combinations (CXI and CTI, each in continuous, quartile, median, and ROC-derived cut-off forms), adjusting for age, tumor stage, carcinomatosis, and malnutrition (Table 5). CTI was eliminated from the model in all four analytical forms, and CXI was similarly eliminated in its continuous, quartile, and median forms. In contrast, CXI dichotomized at the ROC-derived cut-off remained the only form retained in the final model, showing a significant independent association with overall mortality (HR 1.75; 95% CI 1.04–2.95 for low vs. high CXI), alongside advanced tumor stage and malnutrition.

Given the high prevalence of digestive tumors, tumor location was analyzed as digestive vs. other tumors in the overall mortality models (Table S9). Digestive tumors (n = 88; 63%) had lower in-hospital mortality than other tumors (6.8% vs. 19.6%). The association of CXI < 9.03 with in-hospital mortality (OR 4.56 vs. 4.50) and the association of CTI ≥6.71 with ICU admission due to clinical deterioration (OR 4.17 vs. 11.77) was similar in digestive and other tumors (*p* for interaction >0.4), although the number of events per subgroup was small. In contrast, low CXI was associated with overall survival mainly in other tumors (HR 3.43; 95% CI 1.66–7.10) and not in digestive tumors (HR 0.98; 95% CI 0.51–1.88; *p* for interaction = 0.008); the interaction was no longer significant after adjustment for tumor stage and malnutrition (*p* = 0.22).

## 4. Discussion

In the present study, lower CXI values were significantly and consistently associated with in-hospital mortality. Across the full distribution of the index, quartile analysis showed a clear inverse gradient, with in-hospital mortality declining progressively from 20.6% in the lowest quartile to 5.7% in the highest (*p*-trend = 0.029). ROC curve analysis identified an optimal cut-off of 9.03 (AUC 0.69), and patients below this threshold had a higher risk of in-hospital death on univariate analysis (OR 4.74; 95% CI 1.54–14.57). Importantly, when the four analytical forms of CXI—continuous, quartiles, median split, and ROC-derived cut-off—were tested in parallel multivariable models under two independent covariate sets, only the ROC-derived cut-off retained statistical significance with in-hospital mortality (OR 3.91; 95% CI 1.22–12.52; *p* = 0.022). Age, myosteatosis and tumor stage were not retained in the final models, so this estimate is effectively unadjusted. However, with only 14–16 events, these models have limited power to detect confounding, and the association should be interpreted with caution. Taken together, these results point to a threshold-dependent, rather than strictly linear, relationship between CXI and short-term mortality risk, a pattern that is conceptually consistent with the staged nature of cancer cachexia itself, in which clinical and metabolic deterioration accelerates once a critical degree of muscle, nutritional and inflammatory derangement has been reached [1].

These findings are in line with a growing body of evidence supporting the prognostic relevance of CXI in cancer patients. Two recent systematic reviews and meta-analyses encompassing multiple tumor types confirmed that low CXI is consistently associated with worse clinical outcomes, including poorer survival, higher postoperative morbidity, and increased risk of disease progression [10,11]. The meta-analysis by Bas et al. pooled data across a broad spectrum of solid tumors and demonstrated that patients in the low-CXI category faced a significantly higher risk of mortality, reinforcing the index’s value as an integrated marker of nutritional depletion, systemic inflammation, and muscle wasting [10]. Zhang et al., focusing specifically on gastrointestinal cancers, similarly reported that low CXI was independently associated with reduced survival, supporting its applicability beyond the purely surgical context in which it was originally described [11]. These findings have been replicated in disease-specific cohorts including gastric cancer, hepatocellular carcinoma, small-cell lung cancer, and metastatic urothelial carcinoma, among others [15,16,17,18,19]. Most of these studies, however, were conducted in surgical or outpatient settings, or in histologically homogeneous populations, whereas the present cohort comprises a broad range of tumor types evaluated acutely during hospitalization through a structured nutritional-assessment protocol—a clinical scenario that has been comparatively less explored and that may better reflect the complexity of real-world oncological practice.

Hospital readmission was not significantly associated with CXI or CTI in any analytical form among patients discharged alive (quartile trends *p* = 0.083 and *p* = 0.059; median split *p* = 0.302 and *p* = 0.514; ROC cut-offs *p* = 0.579 and *p* = 0.142).

A further, clinically relevant finding was that CXI, when dichotomized at its ROC-derived cut-off, extended its prognostic value beyond the acute hospitalization period to overall survival. While CXI showed no association with overall mortality when analyzed as a continuous variable, in quartiles, or dichotomized by the median—in either Kaplan–Meier or Cox analyses—patients with low CXI defined by the 9.03 threshold had a markedly shorter median survival than those with high CXI (144 vs. 645 days; log-rank *p* = 0.024), and this association remained significant after adjustment for tumor stage, carcinomatosis, and malnutrition in the multivariable Cox model (HR 1.75; 95% CI 1.04–2.95; *p* = 0.035). This finding reconciles our results with several surgical series that have reported an independent association between low CXI and reduced overall survival in esophagogastric and pancreatic cancer [20,21,22], and supports the interpretation that CXI behaves as a threshold marker of a critical degree of cachexia-related derangement—below which the risk of both short- and long-term mortality rises sharply—rather than as a marker with a continuous, dose-dependent relationship with survival.

The association of low CXI with survival was observed mainly in non-digestive tumors (HR 3.43 vs. 0.98 in digestive tumors; *p* for interaction 0.008). The interaction was no longer significant after adjustment for stage and malnutrition (*p* = 0.22), so we cannot confirm that the effect differs by tumor location, and the finding should be considered exploratory.

This pattern should nonetheless be interpreted with appropriate caution. Youden-index-derived cut-offs are optimized within the same dataset used to test their prognostic significance, which can inflate the apparent strength of threshold-based associations relative to their continuous or quartile counterparts—a recognized source of optimism bias in cut-point selection. The CXI cut-off was associated with both in-hospital mortality and overall survival, but these results rely on a small number of events and on a cut-off derived in the same sample, and therefore external validation of the 9.03 cut-off in an independent cohort will be required before it can be adopted as a fixed clinical threshold.

Regarding the CTI, the principal finding was its association with ICU admission. ICU admissions rose across CTI quartiles, from 5.9% in the lowest to 25.7% in the highest (*p*-trend = 0.007). In the multivariable models, CTI remained associated with the outcome at its ROC-derived cut-off of 6.71 regardless of the adjustment set (OR 5.52; 95% CI 1.64–18.54 with surgery and age; OR 4.94; 95% CI 1.48–16.47 with surgery and myosteatosis). The median split and the continuous form gave similar estimates, but they were significant only in some of the models, and the quartiles were eliminated. Surgery was retained in all final models, but 18 of the 19 events occurred in operated patients, so its estimate is imprecise. Restricting the analysis to the 95 operated patients, the association of the CTI cut-off persisted (OR 4.67; 95% CI 1.41–15.48), so it does not seem to be explained by surgery alone. With only 19 events, these results should be considered exploratory.

This association is particularly noteworthy in the context of our cohort, comprising acutely hospitalized cancer patients—a population in whom systemic inflammation and insulin resistance frequently coexist, mutually reinforce one another, and are directly linked to the severity of the acute clinical presentation. A composite index integrating both dimensions may therefore reflect a degree of metabolic derangement that accompanies, and possibly contributes to, acute clinical deterioration and the need for intensive support, beyond what either CRP or the TyG index would convey in isolation. However, the observational design does not allow us to establish causality or temporality.

CXI was also associated with ICU admission due to clinical deterioration in unadjusted analyses: admissions decreased across CXI quartiles (from 26.5% to 2.9%; *p*-trend = 0.005), and the ICU-derived cut-off of 15.79 had an unadjusted OR of 7.69 (95% CI 1.70–34.76). In the multivariable models, however, CXI was eliminated as a continuous variable, in quartiles and as a median split under both adjustment sets, and only the ICU-derived cut-off was retained, with similar estimates in both (OR 5.45; 95% CI 1.18–25.31; *p* = 0.030 with surgery and age; OR 4.56; 95% CI 0.96–21.57; *p* = 0.056 with surgery and myosteatosis). This cut-off is close to the cohort median (13.56) and classifies more than half of the patients as low CXI, so it adds little to the median split and its significance should be interpreted with caution. Both indices showed similar discrimination for this outcome (AUC 0.704 for CTI and 0.721 for CXI), but the association of CTI was more consistent across analytical forms and adjustment sets; given the similar discrimination and the small number of events, this should not be taken as evidence that either index is superior.

These results are consistent with the growing body of evidence supporting the prognostic relevance of CTI in oncological populations. The index was originally proposed and validated by Ruan et al. in a large prospective multicenter cohort, in which higher CTI values were independently associated with reduced overall survival across multiple tumor types [12]. Subsequent prospective work by the same group in gastrointestinal cancer patients confirmed this association, further demonstrating that the prognostic impact of CTI was maintained after adjustment for established clinical and nutritional variables [23]. Its relevance has also been specifically demonstrated in patients with cancer cachexia, where elevated CTI was associated with worse nutritional status and poorer outcomes [13], and in population-based analyses linking higher CTI to increased cancer-specific mortality [24]. However, most of these studies were conducted in outpatient or post-surgical settings and used overall survival as their primary endpoint—a context that differs substantially from ours. To our knowledge, the association of CTI and CXI with ICU admission due to clinical deterioration in acutely hospitalized cancer patients has not been previously reported, and may represent a signal specific to the acute phase of hospital care.

In contrast to CXI, CTI was not significantly associated with overall survival in any of its four analytical forms—continuous, quartile, median, or ROC-derived cut-off—in either Kaplan–Meier or Cox regression analyses. This divergence reinforces a pattern observed throughout the present analyses: while the empirically derived ROC cut-off consistently captured the prognostic signal of CXI more effectively than its continuous, quartile, or median-based forms, and this signal extended from the acute hospitalization period all the way to overall survival, the association of CTI in this cohort appears confined to the acute phase of hospitalization—specifically, ICU admission— without extending to long-term outcomes. This contrasts with the original validation studies of CTI, which were conducted in prospective, largely ambulatory cohorts using overall survival as the primary endpoint [12,13,24]. The discrepancy most likely reflects the dominant prognostic weight of tumor-related factors—advanced disease stage, malnutrition, and carcinomatosis—over long-term follow-up in an acutely hospitalized population such as ours, together with the possibility that CTI, being predominantly a marker of acute systemic inflammation and insulin resistance, is more sensitive to short-term physiological decompensation than to the cumulative, multifactorial determinants of long-term cancer mortality.

Identifying patients at higher risk does not by itself clarify the clinical problems that contribute to their vulnerability, which is particularly relevant in a cohort with a high prevalence of malnutrition (79.0% by GLIM criteria). Standardized nursing diagnoses could help describe the care needs of these patients: in a recent series of 682 inpatients with lung cancer, nutritional deficit and impaired physical mobility were documented in 28.2% and 21.0% of them, respectively [25]. Future studies should investigate whether standardized nursing diagnoses can help characterize the clinical needs of cancer patients with unfavorable cachexia-related indices, particularly regarding nutritional impairment and other coexisting care problems.

Overall, these findings support partly distinct clinical roles for CXI and CTI in hospitalized cancer patients. CXI, particularly when dichotomized at an empirically derived threshold, was associated with both short-term in-hospital death and long-term mortality, and may be most useful as an early marker of a critical degree of cachexia-related muscle and nutritional depletion. Both indices showed similar discrimination for ICU admission due to clinical deterioration, but CTI remained associated with it in more analytical forms than CXI in the adjusted models, whereas CTI showed no association with long-term prognosis in this setting. Because the timing of the assessment relative to surgery and to ICU admission could not be verified in all patients, these findings should be read as associations observed during the index admission and not as predictions. Both indices are readily calculable from routinely available clinical and laboratory data, supporting their potential use for early risk stratification; however, the reliance on ROC-derived cut-offs optimized within a relatively small, single-center cohort—together with the limited number of in-hospital deaths and of ICU admissions due to clinical deterioration—underscores the need for prospective, multicenter validation before these thresholds can be adopted into routine clinical decision-making.

This study has several limitations. First, it is a retrospective, single-center study including only patients evaluated by the Nutrition Unit, which limits its generalizability. Second, the number of events was small (16 in-hospital deaths and 19 ICU admissions due to clinical deterioration), which restricted the number of covariates in the multivariable models. Tumor stage was missing in 15 patients, who were excluded from the mortality models. Third, the ROC-derived cut-offs (CXI < 9.03 for in-hospital mortality; CTI ≥ 6.71 and CXI ≤ 15.79 for ICU admission) were obtained in the same sample without external validation, so their effects are probably overestimated and should be considered exploratory. Fourth, 18 of the 19 ICU admissions occurred in operated patients, so the results cannot be extrapolated to non-surgical patients. Finally, tumor heterogeneity, the small size of the subgroups, and the lack of data on anorexia, food intake and limitation of therapeutic effort may also have influenced the results. Anorexia and reduced food intake are major drivers of cancer cachexia and malnutrition and may themselves influence both the body composition and inflammatory markers that make up these indices and the clinical outcomes studied; as appetite and dietary intake were not recorded, we cannot exclude that they partly explain, or confound, the associations observed, and future prospective studies should systematically assess them.

## 5. Conclusions

In this cohort, CXI and CTI showed complementary, threshold-dependent prognostic values. CXI, at its ROC-derived cut-off (<9.03), was associated with in-hospital mortality (OR 3.91) and, independently of tumor stage, carcinomatosis and malnutrition, with overall survival (HR 1.75). CTI, at its ROC-derived cut-off (≥6.71), was associated with ICU admission due to clinical deterioration after adjustment for surgery and age (OR 5.52) or surgery and myosteatosis (OR 4.94); it was more consistently associated than CXI, whose association was limited to its ICU-derived cut-off (≤15.79; OR 5.45 and 4.56), whereas CTI was not associated with survival. Both indices are readily calculable from routine data and may support early risk stratification, although the single-center design, the small number of events and the uncertain timing of the assessment relative to the outcomes warrant prospective, multicenter validation before clinical adoption.

## Figures and Tables

**Table 1:** Association between CXI and CTI (continuous, quartiles, and median split) and clinical outcomes.

Variable	ICU Admission, *p* (Yes n = 19; No n = 120)	In-Hospital Mortality, *p* (Yes n = 16; No n = 123)	Overall Mortality, *p* (Yes n = 74; No n = 65)
CXI—continuous, median [IQR]	0.002 *	0.013 *	0.460
CXI—quartiles (Q1–Q4)	0.005 *	0.029 *	0.785
CXI—median split (low vs. high)	0.024 *	0.036 *	0.928
CTI—continuous, median [IQR]	0.004 *	0.095	0.253
CTI—quartiles (Q1–Q4)	0.007 *	0.063	0.302
CTI—median split (low vs. high)	0.007 *	0.104	0.803

* *p* < 0.05, statistically significant. n = number of patients in each outcome group (yes vs. no). *p* values compare outcome groups for each analytical form of CXI and CTI: Mann–Whitney U test for continuous values; linear-by-linear association chi-square test (*p* for trend) for quartiles; Pearson chi-square test for the median split. Detailed group-level values (medians [IQR], N (%) by category, for each outcome) are provided in Tables S3–S5 in Supplementary Materials.

**Table 2:** Multivariable logistic regression models (backward Wald) for CXI and in-hospital mortality under two independent adjustment sets.

CXI Form	Adjustment 1: Age + Stage			Adjustment 2: Myosteatosis + Stage			Variables Retained in the Final Model
	OR *	95% CI	*p*	OR *	95% CI	*p*	
Continuous (per 1-unit increase)	1.002	0.989–1.015	0.778	1.002	0.990–1.014	0.740	None
Quartiles (Q1 vs. Q4)	3.15	0.56–17.76	0.193	3.15	0.56–17.76	0.193	None
Median split (low vs. high)	2.73	0.81–9.25	0.105	2.73	0.81–9.25	0.105	None
ROC-derived cut-off (low vs. high)	3.91	1.22–12.52	0.022 †	3.91	1.22–12.52	0.022 †	CXI cut-off only

† *p* < 0.05. * OR expressed as low CXI vs. high CXI throughout. For the continuous, quartile and median forms, the estimate shown is the one from the last step before CXI was removed by the backward procedure (not an independent predictor); these forms and all covariates (age, myosteatosis and tumor stage) were eliminated. Quartiles: global *p* = 0.399 (set 1) and 0.399 (set 2); Q2 and Q3 vs. Q4 in Table S7. Tumor stage was missing in 15 patients, who were excluded from all models. Nagelkerke R^2^ = 0.086 for the final model with the ROC-derived cut-off in both sets. The full estimates of all variables entered are given in Table S7. N = 124 and 14 deaths in all models.

**Table 3:** Multivariable logistic regression models (backward Wald) for CTI and CXI and ICU admission, under two independent adjustment sets.

Index Form	Adjustment 1: Age + Surgery			Adjustment 2: Myosteatosis + Surgery			Variables Retained in the Final Model
	OR *	95% CI	*p*	OR *	95% CI	*p*	
**CTI**							
Continuous (per 1-unit increase)	2.46	0.97–6.24	0.057	2.21	0.90–5.42	0.082	Set 1: CTI, surgery, age Set 2: CTI, surgery, myosteatosis
Quartiles (Q4 vs. Q1)	3.09	0.56–16.96	0.194	2.75	0.50–15.23	0.246	Set 1: surgery, age (no index) Set 2: surgery, myosteatosis (no index)
Median split (high vs. low)	3.43	1.03–11.43	0.045 †	3.14	0.94–10.49	0.063	Set 1: CTI, surgery, age Set 2: CTI, surgery, myosteatosis
ROC-derived cut-off (≥6.71 vs. <6.71)	5.52	1.64–18.54	0.006 †	4.94	1.48–16.47	0.009 †	Set 1: CTI, surgery, age Set 2: CTI, surgery, myosteatosis
**CXI**							
Continuous (per 1-unit increase)	0.966	0.922–1.012	0.144	0.966	0.921–1.014	0.158	Set 1: surgery, age (no index) Set 2: surgery, myosteatosis (no index)
Quartiles (Q1 vs. Q4)	6.11	0.68–55.16	0.107	5.56	0.61–50.43	0.127	Set 1: surgery, age (no index) Set 2: surgery, myosteatosis (no index)
Median split (low vs. high)	1.96	0.61–6.31	0.258	2.17	0.70–6.74	0.181	Set 1: surgery, age (no index) Set 2: surgery, myosteatosis (no index)
ROC-derived cut-off (≤15.79 vs. >15.79)	5.45	1.18–25.31	0.030 †	4.56	0.96–21.57	0.056	Set 1: CXI, surgery Set 2: CXI, surgery, myosteatosis

† *p* < 0.05. * OR expressed as high CTI vs. low CTI and low CXI vs. high CXI throughout (quartiles: Q4 vs. Q1 for CTI and Q1 vs. Q4 for CXI). When the index was eliminated by the backward procedure (not an independent predictor), the estimate shown is the one from the last step before its removal; the covariates retained in each final model are listed in the last column (set 1: surgery + age; set 2: surgery + myosteatosis). Quartiles: global *p* = 0.169 (set 1) and 0.268 (set 2) for CTI, and 0.353 and 0.366 for CXI; Q2 and Q3 vs. the reference quartile in Table S8. All models: N = 139 patients and 19 ICU admissions due to clinical deterioration. Nagelkerke R^2^ of the final models: CTI cut-off 0.267 (set 1) and 0.283 (set 2); CXI cut-off 0.193 (set 1) and 0.248 (set 2). The cut-offs for ICU admission (CTI ≥ 6.71; CXI ≤ 15.79) were derived in the same sample and are exploratory. The full estimates of all variables entered are given in Table S8.

**Table 4:** Kaplan–Meier survival analysis for CXI and CTI, across three analytical forms of each index; N = 139; 74 deaths (53.2%); median follow-up 553 days. Log-rank (Mantel–Cox) test used for group comparisons.

Index	Analytical Form	Log-Rank χ^2^ (*df*)	*p*	Median Survival (Days)
**CXI**				
	Quartiles	1.746 (3)	0.627	Q1 = 144, Q2 = 553, Q3 = 636, Q4 = 574
	Median split	0.335 (1)	0.563	Low = 412, High = 636
	ROC-derived cut-off (9.03)	5.130 (1)	0.024 *	Low = 144, High = 645
**CTI**				
	Quartiles	3.075 (3)	0.380	Q1 = 279, Q2 = 670, Q3 = 553, Q4 = 474
	Median split	0.004 (1)	0.952	Low = 409, High = 553
	ROC-derived cut-off (6.71)	0.045 (1)	0.833	Low = 553, High = 529

* *p* < 0.05, statistically significant. Only CXI dichotomized at its ROC-derived cut-off (9.03) showed a significant difference in overall survival, with markedly shorter median survival in the low-CXI group. See Figure 2 for the corresponding survival curves.

**Table 5:** Multivariable Cox proportional hazards models (backward LR) for CXI and CTI and overall mortality, across four analytical forms of each index.

**Analytical form of CXI**	HR *	**95% CI**	***p***
Continuous (per 1-unit increase)	1.003	0.997–1.009	0.396
Quartiles (Q4 vs. Q2, high vs. low)	0.92	0.45–1.87	0.810
Median split (low vs. high)	1.00	0.60–1.69	0.990
ROC-derived cut-off (low vs. high)	1.75	1.04–2.95	0.035 *
**Analytical form of CTI**	HR *	**95% CI**	***p***
Continuous (per 1-unit increase)	0.97	0.72–1.31	0.825
Quartiles (Q1 vs. Q4, low vs. high)	1.03	0.52–2.03	0.928
Median split (high vs. low)	0.81	0.49–1.35	0.412
ROC-derived cut-off (high vs. low)	1.27	0.75–2.15	0.371

* *p* < 0.05, statistically significant. HR values correspond to the last step before each variable was removed by the backward likelihood-ratio procedure (continuous and median forms of CXI eliminated at step 2; quartile and ROC forms of CTI eliminated at step 3), oriented consistently: CXI as low vs. high, CTI as high vs. low—inverted from the raw SPSS output where needed. Only the ROC-derived cut-off of CXI remained significant and was retained in the final adjustment model; all other forms shown did not survive backward elimination and are reported here for transparency, not as independent predictors.

## Data Availability

The data presented in this study are available on request from the corresponding author due to privacy/ethical restrictions related to patient data.

## Supplementary Materials

The following supporting information can be downloaded at https://www.mdpi.com/article/10.3390/nu18213705/s1; Table S1: Baseline characteristics of the study population; Table S2: (A) Baseline characteristics according to Cachexia Index (CXI) quartiles; (B) Baseline characteristics according to CRP–Triglyceride–Glucose Index (CTI) quartiles; Table S3: Association of CXI and CTI (continuous, quartiles, and median split) with ICU admission; Table S4: Association of CXI and CTI (continuous, quartiles, and median split) with in-hospital mortality; Table S5: Association of CXI and CTI (continuous, quartiles, and median split) with overall mortality; Table S6; Classification (2 × 2) tables of the ROC-derived cut-offs; Table S7: Backward Wald logistic regression models for CXI and in-hospital mortality; Table S8: Backward Wald logistic regression models for CTI and CXI and ICU admission; Table S9: Associations of CXI and CTI with the main outcomes in digestive and other tumors.

## Abbreviations

The following abbreviations are used in this manuscript:

AUC	Area under the curve
BMI	Body mass index
CEICA	Comité de Ética de la Investigación de Aragón
CI	Confidence interval
CRP	C-reactive protein
CT	Computed tomography
CTI	CRP–triglyceride–glucose index
CXI	Cachexia Index
GLIM	Global Leadership Initiative on Malnutrition
HR	Hazard ratio
HU	Hounsfield Units
ICU	Intensive care unit
IQR	Interquartile range
L3	Third lumbar vertebra
MUST	Malnutrition Universal Screening Tool
NLR	Neutrophil-to-lymphocyte ratio
OR	Odds ratio
ROC	Receiver operating characteristic
SARC-F	Strength, Assistance walking, Rise from a chair, Climb stairs, Falls questionnaire
SD	Standard deviation
SMI	Skeletal muscle index
SPSS	Statistical Package for the Social Sciences
STROBE	Strengthening the Reporting of Observational Studies in Epidemiology
TyG	Triglyceride–glucose index
