# Supplementary material for: Cachexia Index and CRP–Triglyceride–Glucose Index as Prognostic Predictors in Cancer Hospitalized Patients
Source: Nutrients. 2026 Nov 8;18(21):3705. doi: 10.3390/nu18213705 (PMC13765414; doi:10.3390/nu18213705)
Supplement: Supplementary file 1 [file nutrients-18-03705-s001.zip › nutrients-4607930-supplementary.pdf]

Table S1. Baseline characteristics of the study population (N=139).

**Variable**	**Overall cohort (N=139)**
Age, years, mean ± SD	65.1 ± 12.7
Sex, male, n (%)	73 (52.5%)
BMI, kg/m², median (IQR)	24.0 (21.1–26.6)
Malnutrition (GLIM), yes, n (%)	98 (79.0%)
**Tumor location, n (%)**	
Digestive	88 (63.3%)
ENT	5 (3.6%)
Hematologic	7 (5.0%)
Gynecologic	12 (8.6%)
Lung	9 (6.5%)
Other	18 (12.97%)
Carcinomatosis, yes, n (%)	34 (25.2%)
**Stage, n (%)**	
Stage I	13 (10.5%)
Stage II	13 (10.5%)
Stage III	27 (21.8%)
Stage IV	71 (57.3%)
**Treatment, yes, n (%)**	
Surgery	95 (68.3%)
Immunotherapy	46 (33.3%)
Chemotherapy	98 (70.5%)
Radiotherapy	30 (21.6%)
Hemoglobin, g/dL, mean ± SD	10.6 ± 1.8
Creatinine, mg/dL, median (IQR)	0.64 (0.48–0.80)
Albumin, g/dL, median (IQR)	2.22 (1.71–2.79)
C-reactive protein, mg/L, median (IQR)	85.3 (28.0–148.1)
NLR, median (IQR)	6.5 (3.3–11.2)
SMI, cm²/m², mean ± SD	40.5 ± 8.9
Muscle radiodensity, HU, mean ± SD	33.5 ± 13.0
Sarcopenia (low SMI), yes, n (%)	111 (79.9%)
Myosteatosis, yes, n (%)	94 (67.6%)
ICU admission due to clinical deterioration, yes, n (%)	19 (13.7%)
Readmission†, yes, n (%)	80 (65.0%)
In-hospital mortality, yes, n (%)	16 (11.5%)
Overall mortality, yes, n (%)	74 (53.2%)
CXI, median (IQR)	13.5 (6.4–27.7)
CTI, median (IQR)	6.61 (6.09–7.05)

Data are presented as n (%) for categorical variables, mean ± SD for normally distributed continuous variables (Kolmogorov-Smirnov p>0.05), and median (interquartile range) otherwise, as indicated for each variable. Percentages are calculated over patients with available data. Missing data: tumor stage, n=15 (percentages calculated over 124 patients); malnutrition according to GLIM, n=15 (over 124 patients); carcinomatosis, n=4 (over 135 patients); immunotherapy, n=1 (over 138 patients). † Calculated among patients discharged alive (n=123). BMI, body mass index; CTI, CRP–triglyceride–glucose index; CXI, cachexia index; ENT, ear, nose and throat; GLIM, Global Leadership Initiative on Malnutrition; HU, Hounsfield units; ICU, intensive care unit; NLR, neutrophil-to-lymphocyte ratio; SMI, skeletal muscle index.

Table S2 (A). Baseline characteristics according to Cachexia Index (CXI) quartiles.

Variable	Q1 (n=34) *CXI <6.43*	Q2 (n=35) *CXI 6.43–13.58*	Q3 (n=35) *CXI 13.59–28.15*	Q4 (n=35) *CXI >28.15*	Total (n=139)	p-trend
Age (years), mean ± SD	68.2 ± 13.9	68.1 ± 9.9	61.1 ± 12.2	63.2 ± 13.3	65.1 ± 12.7	0.023*
BMI (kg/m²), mean ± SD	24.1 ± 4.4	25.8 ± 6.3	23.1 ± 4.2	23.7 ± 3.0	24.2 ± 4.7	0.299
**Laboratory parameters**						
Albumin (g/dL), mean ± SD	1.88 ± 0.63	1.98 ± 0.47	2.45 ± 0.59	3.02 ± 0.72	2.33 ± 0.75	<0.001*
CRP (mg/L), mean ± SD	128.1 ± 82.7	130.9 ± 93.7	94.8 ± 76.1	52.1 ± 80.8	101.3 ± 88.6	<0.001*
Hb (g/dL), mean ± SD	9.85 ± 1.56	10.22 ± 1.43	10.67 ± 1.74	11.45 ± 1.91	10.55 ± 1.76	<0.001*
NLR, mean ± SD	22.4 ± 14.7	8.9 ± 3.3	5.4 ± 1.6	2.1 ± 1.2	9.6 ± 10.7	<0.001*
TyG, mean ± SD	4.86 ± 0.25	4.94 ± 0.29	4.86 ± 0.43	4.71 ± 0.28	4.84 ± 0.33	0.034*
**Body composition**						
SMI (cm²/m²), mean ± SD	36.4 ± 7.9	41.0 ± 9.1	40.9 ± 8.7	43.7 ± 8.6	40.5 ± 8.9	0.001*
Muscle radiodensity (HU), mean ± SD	28.1 ± 14.1	31.0 ± 14.4	36.3 ± 8.6	38.3 ± 12.0	33.5 ± 13.0	<0.001*
**Oncological characteristics**						
Tumor location, N (%)						0.170
Digestive	20 (58.8%)	22 (62.9%)	21 (60.0%)	25 (71.4%)	88 (63.3%)	
ENT	2 (5.9%)	2 (5.7%)	0 (0.0%)	1 (2.9%)	5 (3.6%)	
Hematologic	2 (5.9%)	0 (0.0%)	4 (11.4%)	1 (2.9%)	7 (5.0%)	
Gynecologic	2 (5.9%)	1 (2.9%)	7 (20.0%)	2 (5.7%)	12 (8.6%)	
Lung	2 (5.9%)	3 (8.6%)	2 (5.7%)	2 (5.7%)	9 (6.5%)	
Other (incl. urothelial)	6 (17.6%)	7 (20.0%)	1 (2.9%)	4 (11.4%)	18 (12.9%)	
Advanced stage III-IV, N (%)	21 (75.0%)	29 (85.3%)	25 (78.1%)	24 (77.4%)	99 (79.2%)	0.956
Carcinomatosis, N (%)	4 (11.8%)	12 (34.3%)	9 (25.7%)	9 (25.7%)	34 (24.5%)	0.320
**Oncological treatment**						
Surgery, N (%)	28 (82.4%)	27 (77.1%)	25 (71.4%)	15 (42.9%)	95 (68.3%)	<0.001*
Chemotherapy, N (%)	20 (58.8%)	24 (68.6%)	25 (71.4%)	29 (82.9%)	98 (70.5%)	0.031*
Radiotherapy, N (%)	7 (20.6%)	7 (20.0%)	9 (25.7%)	7 (20.0%)	30 (21.6%)	0.901
Immunotherapy, N (%)	8 (23.5%)	14 (40.0%)	16 (45.7%)	8 (22.9%)	46 (33.1%)	0.935
**Nutritional status**						
Malnutrition (GLIM), N (%)	27 (93.1%)	24 (75.0%)	22 (71.0%)	25 (78.1%)	98 (79.0%)	0.156
Sarcopenia, N (%)	30 (88.2%)	28 (80.0%)	25 (71.4%)	28 (80.0%)	111 (79.9%)	0.281
Myosteatosis, N (%)	27 (79.4%)	25 (71.4%)	23 (65.7%)	19 (54.3%)	94 (67.6%)	0.023*
**Clinical outcomes**						
ICU admission, N (%)	9 (26.5%)	5 (14.3%)	4 (11.4%)	1 (2.9%)	19 (13.7%)	0.005*
Readmission†, N (%)	16 (59.3%)	18 (60.0%)	19 (57.6%)	27 (81.8%)	80 (65.0%)	0.083
Overall mortality, N (%)	20 (58.8%)	16 (45.7%)	20 (57.1%)	18 (51.4%)	74 (53.2%)	0.785
In-hospital mortality, N (%)	7 (20.6%)	5 (14.3%)	2 (5.7%)	2 (5.7%)	16 (11.5%)	0.029*
**Indices**						
CTI, mean ± SD	6.74 ± 0.48	6.77 ± 0.63	6.52 ± 0.76	5.81 ± 0.84	6.46 ± 0.79	<0.001*

† Calculated among patients discharged alive (n=123; Q1 n=27, Q2 n=30, Q3 n=33, Q4 n=33). * p<0.05, statistically significant. p-trend for continuous variables: linear regression with quartile as an ordinal predictor. p-trend for categorical variables: linear-by-linear association chi-square.

Table S2 (B). Baseline characteristics according to CRP–Triglyceride–Glucose Index (CTI) quartiles.

Variable	Q1 (n=34) *CTI <6.09*	Q2 (n=35) *CTI 6.09–6.61*	Q3 (n=35) *CTI 6.62–7.05*	Q4 (n=35) *CTI >7.05*	Total (n=139)	p-trend
Age (years), mean ± SD	63.9 ± 13.1	65.1 ± 15.3	65.0 ± 11.9	66.4 ± 10.2	65.1 ± 12.7	0.441

Variable	Q1 (n=34) *CTI <6.09*	Q2 (n=35) *CTI 6.09–6.61*	Q3 (n=35) *CTI 6.62–7.05*	Q4 (n=35) *CTI >7.05*	Total (n=139)	p-trend
BMI (kg/m²), mean ± SD	22.9 ± 4.5	23.4 ± 4.4	25.3 ± 4.7	25.0 ± 4.9	24.2 ± 4.7	0.020*
**Laboratory parameters**						
Albumin (g/dL), mean ± SD	2.86 ± 0.68	2.26 ± 0.61	2.13 ± 0.81	2.10 ± 0.67	2.33 ± 0.75	<0.001*
CRP (mg/L), mean ± SD	9.1 ± 10.0	74.5 ± 50.6	131.3 ± 56.5	187.7 ± 90.5	101.3 ± 88.6	<0.001*
Hb (g/dL), mean ± SD	11.62 ± 1.60	10.34 ± 1.56	10.23 ± 2.10	10.06 ± 1.30	10.55 ± 1.76	0.009*
NLR, mean ± SD	3.99 ± 4.63	9.90 ± 10.14	12.10 ± 11.48	12.22 ± 12.83	9.59 ± 10.70	0.001*
TyG, mean ± SD	4.65 ± 0.30	4.68 ± 0.26	4.85 ± 0.17	5.19 ± 0.27	4.84 ± 0.33	<0.001*
**Body composition**						
SMI (cm²/m²), mean ± SD	37.8 ± 7.9	41.0 ± 9.0	40.8 ± 8.3	42.5 ± 9.9	40.5 ± 8.9	0.039*
Muscle radiodensity (HU), mean ± SD	37.6 ± 11.4	34.6 ± 12.3	31.6 ± 16.3	30.1 ± 10.2	33.5 ± 13.0	0.009*
**Oncological characteristics**						
Tumor location, N (%)						0.974
Digestive	21 (61.8%)	23 (65.7%)	22 (62.9%)	22 (62.9%)	88 (63.3%)	
ENT	1 (2.9%)	2 (5.7%)	2 (5.7%)	0 (0.0%)	5 (3.6%)	
Hematologic	2 (5.9%)	2 (5.7%)	1 (2.9%)	2 (5.7%)	7 (5.0%)	
Gynecologic	4 (11.8%)	2 (5.7%)	2 (5.7%)	4 (11.4%)	12 (8.6%)	
Lung	2 (5.9%)	1 (2.9%)	4 (11.4%)	2 (5.7%)	9 (6.5%)	
Other (incl. urothelial)	4 (11.8%)	5 (14.3%)	4 (11.4%)	5 (14.3%)	18 (12.9%)	
Advanced stage III-IV, N (%)	26 (81.3%)	29 (87.9%)	22 (75.9%)	22 (71.0%)	99 (79.2%)	0.188
Carcinomatosis, N (%)	10 (29.4%)	6 (17.1%)	9 (25.7%)	9 (25.7%)	34 (24.5%)	0.948
**Oncological treatment**						
Surgery, N (%)	15 (44.1%)	25 (71.4%)	28 (80.0%)	27 (77.1%)	95 (68.3%)	0.003*
Chemotherapy, N (%)	26 (76.5%)	22 (62.9%)	24 (68.6%)	26 (74.3%)	98 (70.5%)	0.992
Radiotherapy, N (%)	10 (29.4%)	8 (22.9%)	5 (14.3%)	7 (20.0%)	30 (21.6%)	0.244
Immunotherapy, N (%)	12 (35.3%)	8 (22.9%)	15 (42.9%)	11 (31.4%)	46 (33.1%)	0.809
**Nutritional status**						
Malnutrition (GLIM), N (%)	26 (76.5%)	25 (78.1%)	24 (80.0%)	23 (82.1%)	98 (79.0%)	0.567
Sarcopenia, N (%)	29 (85.3%)	28 (80.0%)	26 (74.3%)	28 (80.0%)	111 (79.9%)	0.485
Myosteatosis, N (%)	21 (61.8%)	23 (65.7%)	21 (60.0%)	29 (82.9%)	94 (67.6%)	0.106
**Clinical outcomes**						
ICU admission, N (%)	2 (5.9%)	2 (5.7%)	6 (17.1%)	9 (25.7%)	19 (13.7%)	0.007*
Readmission†, N (%)	24 (80.0%)	20 (58.8%)	23 (69.7%)	13 (50.0%)	80 (65.0%)	0.059
Overall mortality, N (%)	23 (67.6%)	15 (42.9%)	18 (51.4%)	18 (51.4%)	74 (53.2%)	0.302
In-hospital mortality, N (%)	4 (11.8%)	1 (2.9%)	2 (5.7%)	9 (25.7%)	16 (11.5%)	0.063
**Indices**						
CXI, mean ± SD	54.1 ± 42.1	22.0 ± 32.0	16.7 ± 23.7	56.6 ± 219.8	37.2 ± 114.2	0.966

† Calculated among patients discharged alive (n=123; Q1 n=30, Q2 n=34, Q3 n=33, Q4 n=26). * p<0.05, statistically significant. p-trend for continuous variables: linear regression with quartile as an ordinal predictor. p-trend for categorical variables: linear-by-linear association chi-square.

Table S3. Association of CXI and CTI (continuous, quartiles, and median split) with ICU admission.

Variable	ICU admission — No (N=120)	ICU admission — Yes (N=19)	p
CXI — continuous variable, median [IQR]			
CXI, median [IQR]	14.66 [6.81–39.10]	6.48 [1.97–14.33]	0.002*
CXI — quartiles, N (%)			

Variable	ICU admission — No (N=120)	ICU admission — Yes (N=19)	p
Q1	25 (20.8%)	9 (47.4%)	0.005*
Q2	30 (25.0%)	5 (26.3%)	
Q3	31 (25.8%)	4 (21.1%)	
Q4	34 (28.3%)	1 (5.3%)	
CXI — median split (low/high), N (%)			
Low CXI (<median)	55 (45.8%)	14 (73.7%)	0.024*
High CXI (≥median)	65 (54.2%)	5 (26.3%)	
CTI — continuous variable, median [IQR]			
CTI, median [IQR]	6.54 [5.93–6.99]	7.02 [6.71–7.25]	0.004*
CTI — quartiles, N (%)			
Q1	32 (26.7%)	2 (10.5%)	0.007*
Q2	33 (27.5%)	2 (10.5%)	
Q3	29 (24.2%)	6 (31.6%)	
Q4	26 (21.7%)	9 (47.4%)	
CTI — median split (low/high), N (%)			
Low CTI (<median)	65 (54.2%)	4 (21.1%)	0.007*
High CTI (≥median)	55 (45.8%)	15 (78.9%)	

* p<0.05, statistically significant. p values: Mann–Whitney U test (continuous CXI/CTI); linear-by-linear association chi-square test, i.e., p for trend (quartiles); Pearson chi-square test (median split). CXI median = 13.56; CTI median = 6.62.

Table S4. Association of CXI and CTI (continuous, quartiles, and median split) with in-hospital mortality.

Variable	In-hospital mortality — No (N=123)	In-hospital mortality — Yes (N=16)	p
CXI — continuous variable, median [IQR]			
CXI, median [IQR]	14.65 [6.77–38.93]	6.59 [3.42–11.65]	0.013*
CXI — quartiles, N (%)			
Q1	27 (22.0%)	7 (43.8%)	0.029*
Q2	30 (24.4%)	5 (31.3%)	
Q3	33 (26.8%)	2 (12.5%)	
Q4	33 (26.8%)	2 (12.5%)	
CXI — median split (low/high), N (%)			
Low CXI (<median)	58 (47.2%)	12 (75.0%)	0.036*
High CXI (≥median)	65 (52.8%)	4 (25.0%)	
CTI — continuous variable, median [IQR]			
CTI, median [IQR]	6.60 [6.09–7.00]	7.06 [6.09–7.13]	0.095
CTI — quartiles, N (%)			
Q1	30 (24.4%)	4 (25.0%)	0.063
Q2	34 (27.6%)	1 (6.3%)	
Q3	33 (26.8%)	2 (12.5%)	
Q4	26 (21.1%)	9 (56.3%)	
CTI — median split (low/high), N (%)			
Low CTI (<median)	65 (52.8%)	5 (31.3%)	0.104
High CTI (≥median)	58 (47.2%)	11 (68.8%)	

* p<0.05, statistically significant. p values: Mann–Whitney U test (continuous CXI/CTI); linear-by-linear association chi-square test, i.e., p for trend (quartiles); Pearson chi-square test (median split). CXI median = 13.56; CTI median = 6.62.Caution is warranted given the low number of in-hospital deaths (N=16), with expected cell counts <5 in the CTI quartile analysis.

Table S5. Association of CXI and CTI (continuous, quartiles, and median split) with overall mortality.

Variable	Overall mortality — No (N=65)	Overall mortality — Yes (N=74)	p
CXI — continuous variable, median [IQR]			
CXI, median [IQR]	13.33 [6.97–28.24]	13.78 [5.13–26.05]	0.460
CXI — quartiles, N (%)			
Q1	14 (21.5%)	20 (27.0%)	0.785
Q2	19 (29.2%)	16 (21.6%)	
Q3	15 (23.1%)	20 (27.0%)	
Q4	17 (26.2%)	18 (24.3%)	
CXI — median split (low/high), N (%)			
Low CXI (<median)	33 (50.8%)	37 (50.0%)	0.928
High CXI (≥median)	32 (49.2%)	37 (50.0%)	
CTI — continuous variable, median [IQR]			
CTI, median [IQR]	6.63 [6.27–7.06]	6.55 [5.80–7.01]	0.253
CTI — quartiles, N (%)			
Q1	11 (16.9%)	23 (31.1%)	0.302
Q2	20 (30.8%)	15 (20.3%)	
Q3	17 (26.2%)	18 (24.3%)	
Q4	17 (26.2%)	18 (24.3%)	
CTI — median split (low/high), N (%)			
Low CTI (<median)	32 (49.2%)	38 (51.4%)	0.803
High CTI (≥median)	33 (50.8%)	36 (48.6%)	

* p<0.05, statistically significant. p values: Mann–Whitney U test (continuous CXI/CTI); linear-by-linear association chi-square test, i.e., p for trend (quartiles); Pearson chi-square test (median split). CXI median = 13.56; CTI median = 6.62..

Table S6. Classification (2×2) tables of the ROC-derived cut-offs.

(a) CXI <9.03 and in-hospital mortality

	In-hospital death	Alive at discharge	Total
CXI <9.03	11	39	50
CXI ≥9.03	5	84	89
Total	16	123	139

Sensitivity 68.8%; specificity 68.3%.

(b) CTI ≥6.71 and ICU admission due to clinical deterioration

	ICU admission	No ICU admission	Total
CTI ≥6.71	15	45	60
CTI <6.71	4	75	79
Total	19	120	139

Sensitivity 78.9%; specificity 62.5%.

(c) CXI ≤15.79 and ICU admission due to clinical deterioration

	ICU admission	No ICU admission	Total
CXI ≤15.79	17	63	80
CXI >15.79	2	57	59
Total	19	120	139

Sensitivity 89.5%; specificity 47.5%.

Rows: test result according to the cut-off; columns: observed outcome. Cut-offs were selected with the Youden index in the same sample (N=139) and are exploratory.

Table S7. Backward Wald logistic regression models for CXI and in-hospital mortality: all variables entered, with the estimates of the full model and the outcome of the elimination procedure (N = 124; 14 events in every model).

Variable	Full model (step 1) OR (95% CI)	p	Final model
**Adjustment 1: age + stage. CXI continuous**			
CXI (per 1-unit increase)	1.002 (0.989–1.015)	0.778	Removed at step 2
Age (per year)	0.99 (0.95–1.04)	0.721	Removed at step 3
Advanced stage III–IV (vs. I–II)	1.72 (0.35–8.41)	0.503	Removed at step 4
**Adjustment 1: age + stage. CXI quartiles**			
Advanced stage III–IV (vs. I–II)	1.65 (0.33–8.13)	0.541	Removed at step 2
CXI quartiles (global test)	—	0.339	Removed at step 4
Quartile Q1 vs. Q4	3.50 (0.61–20.20)	0.161	Removed at step 4
Quartile Q2 vs. Q4	2.69 (0.47–15.54)	0.269	Removed at step 4
Quartile Q3 vs. Q4	0.94 (0.12–7.20)	0.957	Removed at step 4
Age (per year)	0.98 (0.94–1.03)	0.439	Removed at step 3
**Adjustment 1: age + stage. CXI median split**			
Advanced stage III–IV (vs. I–II)	1.57 (0.32–7.63)	0.577	Removed at step 2
Age (per year)	0.98 (0.94–1.03)	0.473	Removed at step 3
CXI low vs. high (median split)	2.95 (0.85–10.29)	0.089	Removed at step 4
**Adjustment 1: age + stage. CXI ROC-derived cut-off (<9.03)**			
Advanced stage III–IV (vs. I–II)	1.70 (0.34–8.40)	0.517	Removed at step 2
Age (per year)	0.98 (0.94–1.03)	0.471	Removed at step 3
CXI ROC cut-off: low vs. high	4.18 (1.28–13.67)	0.018	Retained: 3.91 (1.22–12.52); p=0.022
**Adjustment 2: myosteatosis + stage. CXI continuous**			
Advanced stage III–IV (vs. I–II)	1.68 (0.34–8.19)	0.521	Removed at step 3
Myosteatosis (yes vs. no)	1.83 (0.48–6.98)	0.376	Removed at step 4
CXI (per 1-unit increase)	1.002 (0.990–1.014)	0.740	Removed at step 2
**Adjustment 2: myosteatosis + stage. CXI quartiles**			
Advanced stage III–IV (vs. I–II)	1.65 (0.34–8.07)	0.536	Removed at step 3
Myosteatosis (yes vs. no)	1.54 (0.39–6.12)	0.537	Removed at step 2
CXI quartiles (global test)	—	0.467	Removed at step 4
Quartile Q1 vs. Q4	2.84 (0.48–16.64)	0.248	Removed at step 4
Quartile Q2 vs. Q4	2.27 (0.40–12.84)	0.356	Removed at step 4
Quartile Q3 vs. Q4	0.90 (0.12–6.91)	0.917	Removed at step 4
**Adjustment 2: myosteatosis + stage. CXI median split**			
Advanced stage III–IV (vs. I–II)	1.61 (0.33–7.81)	0.557	Removed at step 2
Myosteatosis (yes vs. no)	1.62 (0.42–6.29)	0.486	Removed at step 3
CXI low vs. high (median split)	2.57 (0.75–8.78)	0.131	Removed at step 4
**Adjustment 2: myosteatosis + stage. CXI ROC-derived cut-off (<9.03)**			
Advanced stage III–IV (vs. I–II)	1.67 (0.34–8.21)	0.531	Removed at step 3
Myosteatosis (yes vs. no)	1.49 (0.38–5.91)	0.566	Removed at step 2
CXI ROC cut-off: low vs. high	3.73 (1.15–12.11)	0.028	Retained: 3.91 (1.22–12.52); p=0.022

Candidate variables entered in each model: CXI in the form indicated, age or myosteatosis (according to the adjustment set) and advanced tumour stage (III–IV vs. I–II). Elimination used the Wald test (probability to enter 0.05, to remove 0.10). "Final model" shows the estimate of the variables still in the model when elimination stopped; variables removed at a given step are not in the final model. In every model, except that for the ROC-derived cut-off, all variables were removed and the final model contained only the constant. Odds ratios are expressed for low vs. high CXI (median split and ROC-

derived cut-off), for advanced vs. non-advanced stage and for presence vs. absence of myosteatosis; quartiles are expressed as Q1, Q2 and Q3 vs. Q4.

Table S8. Backward Wald logistic regression models for CTI and CXI and ICU admission: all variables entered, with the estimates of the full model and the outcome of the elimination procedure (N = 139; 19 events in every model), and sensitivity analysis in operated patients (N = 95; 18 events).

Variable	Full model (step 1) OR (95% CI)	p	Final model
**Adjustment 1: surgery + age. CTI continuous**			
CTI (per 1-unit increase)	2.46 (0.97–6.24)	0.057	Retained: 2.46 (0.97–6.24); p=0.057
Surgery (yes vs. no)	7.32 (0.91–58.67)	0.061	Retained: 7.32 (0.91–58.67); p=0.061
Age (per year)	1.04 (0.99–1.09)	0.098	Retained: 1.04 (0.99–1.09); p=0.098
**Adjustment 1: surgery + age. CTI quartiles**			
CTI quartiles (global test)	—	0.169	Removed at step 2
Quartile Q2 vs. Q1	0.53 (0.06–4.35)	0.553	Removed at step 2
Quartile Q3 vs. Q1	1.76 (0.30–10.32)	0.531	Removed at step 2
Quartile Q4 vs. Q1	3.09 (0.56–16.96)	0.194	Removed at step 2
Surgery (yes vs. no)	8.62 (1.05–70.88)	0.045	Retained: 10.45 (1.33–81.74); p=0.025
Age (per year)	1.04 (1.00–1.10)	0.075	Retained: 1.04 (1.00–1.09); p=0.062
**Adjustment 1: surgery + age. CTI median split**			
CTI high vs. low (median split)	3.43 (1.03–11.43)	0.045	Retained: 3.43 (1.03–11.43); p=0.045
Surgery (yes vs. no)	7.68 (0.96–61.27)	0.054	Retained: 7.68 (0.96–61.27); p=0.054
Age (per year)	1.04 (1.00–1.09)	0.078	Retained: 1.04 (1.00–1.09); p=0.078
**Adjustment 1: surgery + age. CTI ROC-derived cut-off (≥6.71)**			
CTI ROC cut-off: high vs. low	5.52 (1.64–18.54)	0.006	Retained: 5.52 (1.64–18.54); p=0.006
Surgery (yes vs. no)	7.63 (0.95–61.09)	0.056	Retained: 7.63 (0.95–61.09); p=0.056
Age (per year)	1.05 (1.00–1.10)	0.061	Retained: 1.05 (1.00–1.10); p=0.061
**Adjustment 1: surgery + age. CXI continuous**			
CXI (per 1-unit increase)	0.966 (0.922–1.012)	0.144	Removed at step 2
Surgery (yes vs. no)	7.06 (0.88–56.87)	0.066	Retained: 10.45 (1.33–81.74); p=0.025
Age (per year)	1.03 (0.99–1.08)	0.130	Retained: 1.04 (1.00–1.09); p=0.062
**Adjustment 1: surgery + age. CXI quartiles**			
CXI quartiles (global test)	—	0.353	Removed at step 2
Quartile Q1 vs. Q4	6.11 (0.68–55.16)	0.107	Removed at step 2
Quartile Q2 vs. Q4	2.99 (0.31–28.78)	0.344	Removed at step 2
Quartile Q3 vs. Q4	3.26 (0.33–32.30)	0.313	Removed at step 2
Surgery (yes vs. no)	7.29 (0.90–59.09)	0.063	Retained: 10.45 (1.33–81.74); p=0.025
Age (per year)	1.03 (0.99–1.08)	0.145	Retained: 1.04 (1.00–1.09); p=0.062
**Adjustment 1: surgery + age. CXI median split**			
CXI low vs. high (median split)	1.96 (0.61–6.31)	0.258	Removed at step 2
Surgery (yes vs. no)	8.51 (1.06–68.06)	0.044	Retained: 10.45 (1.33–81.74); p=0.025
Age (per year)	1.03 (0.99–1.08)	0.150	Retained: 1.04 (1.00–1.09); p=0.062
**Adjustment 1: surgery + age. CXI ROC-derived cut-off (≤15.79)**			
CXI ROC cut-off: low vs. high	4.65 (0.98–22.08)	0.053	Retained: 5.45 (1.18–25.31); p=0.030
Surgery (yes vs. no)	6.52 (0.80–52.87)	0.079	Retained: 6.52 (0.81–52.27); p=0.077
Age (per year)	1.03 (0.99–1.08)	0.152	Removed at step 2
**Adjustment 2: surgery + myosteatosis. CTI continuous**			
CTI (per 1-unit increase)	2.21 (0.90–5.42)	0.082	Retained: 2.21 (0.90–5.42); p=0.082
Surgery (yes vs. no)	8.36 (1.04–67.20)	0.046	Retained: 8.36 (1.04–67.20); p=0.046
Myosteatosis (yes vs. no)	4.51 (0.96–21.27)	0.057	Retained: 4.51 (0.96–21.27); p=0.057
**Adjustment 2: surgery + myosteatosis. CTI quartiles**			

Variable	Full model (step 1) OR (95% CI)	p	Final model
CTI quartiles (global test)	—	0.268	Removed at step 2
Quartile Q2 vs. Q1	0.59 (0.07–4.75)	0.616	Removed at step 2
Quartile Q3 vs. Q1	1.87 (0.32–11.04)	0.488	Removed at step 2
Quartile Q4 vs. Q1	2.75 (0.50–15.23)	0.246	Removed at step 2
Surgery (yes vs. no)	9.31 (1.14–76.14)	0.037	Retained: 11.13 (1.42–87.21); p=0.022
Myosteatosis (yes vs. no)	4.56 (0.96–21.64)	0.056	Retained: 5.35 (1.16–24.71); p=0.032
**Adjustment 2: surgery + myosteatosis. CTI median split**			
CTI high vs. low (median split)	3.14 (0.94–10.49)	0.063	Retained: 3.14 (0.94–10.49); p=0.063
Surgery (yes vs. no)	8.50 (1.06–68.00)	0.044	Retained: 8.50 (1.06–68.00); p=0.044
Myosteatosis (yes vs. no)	4.77 (1.02–22.35)	0.048	Retained: 4.77 (1.02–22.35); p=0.048
**Adjustment 2: surgery + myosteatosis. CTI ROC-derived cut-off (≥6.71)**			
CTI ROC cut-off: high vs. low	4.94 (1.48–16.47)	0.009	Retained: 4.94 (1.48–16.47); p=0.009
Surgery (yes vs. no)	8.53 (1.06–68.65)	0.044	Retained: 8.53 (1.06–68.65); p=0.044
Myosteatosis (yes vs. no)	4.88 (1.03–23.23)	0.046	Retained: 4.88 (1.03–23.23); p=0.046
**Adjustment 2: surgery + myosteatosis. CXI continuous**			
CXI (per 1-unit increase)	0.966 (0.921–1.014)	0.158	Removed at step 2
Surgery (yes vs. no)	7.85 (0.98–63.14)	0.053	Retained: 11.13 (1.42–87.21); p=0.022
Myosteatosis (yes vs. no)	4.58 (0.97–21.55)	0.054	Retained: 5.35 (1.16–24.71); p=0.032
**Adjustment 2: surgery + myosteatosis. CXI quartiles**			
CXI quartiles (global test)	—	0.366	Removed at step 2
Quartile Q1 vs. Q4	5.56 (0.61–50.43)	0.127	Removed at step 2
Quartile Q2 vs. Q4	2.95 (0.30–28.56)	0.350	Removed at step 2
Quartile Q3 vs. Q4	2.51 (0.25–25.39)	0.434	Removed at step 2
Surgery (yes vs. no)	8.20 (1.02–66.26)	0.048	Retained: 11.13 (1.42–87.21); p=0.022
Myosteatosis (yes vs. no)	4.42 (0.94–20.88)	0.060	Retained: 5.35 (1.16–24.71); p=0.032
**Adjustment 2: surgery + myosteatosis. CXI median split**			
CXI low vs. high (median split)	2.17 (0.70–6.74)	0.181	Removed at step 2
Surgery (yes vs. no)	9.43 (1.18–75.07)	0.034	Retained: 11.13 (1.42–87.21); p=0.022
Myosteatosis (yes vs. no)	4.83 (1.03–22.58)	0.045	Retained: 5.35 (1.16–24.71); p=0.032
**Adjustment 2: surgery + myosteatosis. CXI ROC-derived cut-off (≤15.79)**			
CXI ROC cut-off: low vs. high	4.56 (0.96–21.57)	0.056	Retained: 4.56 (0.96–21.57); p=0.056
Surgery (yes vs. no)	7.74 (0.96–62.66)	0.055	Retained: 7.74 (0.96–62.66); p=0.055
Myosteatosis (yes vs. no)	4.50 (0.95–21.26)	0.058	Retained: 4.50 (0.95–21.26); p=0.058
**Sensitivity analysis in operated patients (N=95; 18 events): index only, unadjusted (enter method)**			
CTI (per 1-unit increase)	2.40 (0.94–6.13)	0.067	No elimination (index only)
CTI quartile Q4 vs. Q1	2.74 (0.50–15.02)	0.246	Global p=0.261
CTI high vs. low (median split)	3.07 (0.93–10.18)	0.066	No elimination (index only)
CTI ≥6.71 vs. <6.71	4.67 (1.41–15.48)	0.012	No elimination (index only)
CXI (per 1-unit increase)	0.963 (0.915–1.013)	0.144	No elimination (index only)
CXI quartile Q1 vs. Q4	6.63 (0.75–58.56)	0.089	Global p=0.201
CXI low vs. high (median split)	2.17 (0.70–6.67)	0.178	No elimination (index only)
CXI ≤15.79 vs. >15.79	4.57 (0.98–21.36)	0.053	No elimination (index only)

Candidate variables entered in each model: the index in the form indicated, surgery, and age or myosteatosis (according to the adjustment set). Probability to enter 0.05 and to remove 0.10. ORs are expressed for high vs. low CTI and low vs. high CXI (median split and ROC-derived cut-offs), for surgery and myosteatosis (yes vs. no), per year of age, and per 1-unit increase for the continuous forms; quartiles are expressed against Q4 (CXI) or Q1 (CTI). "Retained" indicates the estimate of the final model. The cut-offs for ICU admission (CTI ≥6.71; CXI ≤15.79) were derived in the same sample and are exploratory. In the sensitivity analysis the index was the only variable entered, because 18 events do not allow further adjustment.

Table S9. Associations of CXI and CTI with the main outcomes in digestive and other tumors.

	Digestive	Other	p interaction
Patients, n	88 (10 ICU admissions)	51 (9 ICU admissions)	
CXI <9.03 and in-hospital mortality, OR (95% CI)	4.56 (0.78–26.55)	4.50 (1.00–20.15)	>0.5
CTI ≥6.71 and ICU admission, OR (95% CI)	4.17 (1.00–17.40)	11.77 (1.35–102.86)	0.43
CXI <9.03 and overall survival, HR (95% CI)	0.98 (0.51–1.88)	3.43 (1.66–7.10)	0.008

Univariable logistic (OR) and Cox (HR) regression in each group; low vs. high CXI and high vs. low CTI. p interaction: p of the index × tumor group term. For overall survival, the interaction adjusted for tumor stage and malnutrition (n=114) was p=0.22 (HR of low CXI in other tumors 2.42; 95% CI 1.10–5.30). The CXI cut-off ≤15.79 could not be estimated in other tumors, because all 9 events occurred in the low-CXI group.
